# Risk-based early prevention in comparison with routine prevention of dental caries: a 7-year follow-up of a controlled clinical trial; clinical and economic aspects

**DOI:** 10.1186/1472-6831-5-2

**Published:** 2005-03-23

**Authors:** Kaisu Pienihäkkinen, Jorma Jokela, Pentti Alanen

**Affiliations:** 1 Institute of Dentistry, University of Turku Lemminkäisenkatu 2, FI-20520 Turku, Finland; 2 Public Health Care Centre Korpilahti-Muurame 41800 Korpilahti, Finland

## Abstract

**Background:**

The results in an earlier study with 2–5-year-old children indicated that, in comparison with conventional prevention, a risk-based prevention programme was effective in reducing dental caries in a low-caries community. The aim of the present study was to examine the clinical and economic findings seven years after the cessation of the targeted programme, from the perspective of public health care.

**Methods:**

The present material was collected from the dental records of the public health care centres, and included all dental visits after the 5-year examination until the 12-year examination. The groups were compared in relation to clinically detected caries at the age of 12 years, the number of dental visits needed from 5 to 12 years of age, and the estimation of running costs during these years. Statistical analyses included univariate analysis of variance, and calculation of absolute risk reduction and number needed to treat (NNT) values.

**Results:**

At the age of 12 years, DMF was significantly related to the risk category determined ten years earlier, in both study groups. In the risk-based group, the absolute risk reduction for caries in permanent dentition was 0.13 (95% confidence interval 0.06 – 0.21), and the associated NNT value was 8 (95% confidence interval 5 – 17). The total number of preventive, as well as restorative visits was lower in the risk-based than in the routine prevention group. The findings indicate that early risk-based prevention can be correctly targeted, clinically effective, and economically profitable also from the long-term point of view.

**Conclusion:**

Early prevention of dental caries also has long-term benefits in a 7-year follow-up perspective. This seems to hold true as regards targeting, as well as clinical and economic effectiveness. Success in risk-based prevention enables successful work division, and consequently, economic effectiveness.

## Background

The clinical and economic effectiveness of caries prevention cannot be correctly calculated without lengthy follow-ups. A prevention programme leading to postponement of the cavitation process can be favourable if the restorative treatment can be carried out more easily later when the children are older. Some observations and studies, however, suggest that if the timing of the prevention coincides with the eruption of the teeth, the preventive result may remain the same or be even better for several years after the cessation of the preventive programme. Those age cohorts, whose permanent teeth had erupted during the years when sugar consumption was strongly reduced because of the Second World War, still more often had intact teeth than younger cohorts about 40 years later [1]. The same observation was made by Honkala et al. in Karelia [2]. Köhler et al. have reported a long-term preventive effect in children after chlorhexidine treatment of the mother's dentition [3]. Analogously, several field trials with xylitol have indicated that the differences in caries increment figures between the xylitol and control groups increased after the cessation of the use of xylitol, especially in teeth which erupted in the mouth after the implementation of the xylitol programme [4-7].

The explanation for these results may be that the absence of sugar from the diet or the application of prevention before or around the time of eruption of the teeth can give an opportunity for the teeth to erupt and mature in a favourable environment. Therefore, the timing may play a crucial role in the success of caries prevention and, consequently, also in the economic results. Because there is a lack of long-lasting caries prevention trials we are also short of economic studies on the long-term effectiveness of these measures. In addition, a methodological problem is obvious: if the preventive measures are implemented in early childhood with success, the learned habits such as maintenance of good oral hygiene and use of fluorides may remain even after cessation of the intensive prevention programme. In the same way, if the dental professionals are happy with their results, they will pay special attention to the prevention also afterwards. For such reasons, it is quite impossible to reveal the exact role of the applied preventive measures or programmes afterwards or to measure their share in the long-term clinical and economic outcomes.

The results in an earlier study with 2–5-year-old children indicated that, in comparison with conventional prevention, a risk-based prevention programme was effective in reducing dental caries in a low-caries community [8,9]. The screening was based on the presence of mutans streptococci (MS) and incipient carious lesions at the age of two years. MS-negative and caries-free children were considered to be at "low risk", MS-positive but caries-free children at "intermediate risk", and children with any caries lesions at "high risk" of caries. The intensity of the prevention increased with the increasing estimated risk. Basic prevention provided annually to all subjects included dental health education to parents on oral hygiene, fluoride toothpaste, and sweets. In the intermediate-risk category, the children additionally received fluoride varnish treatments twice a year. In the high-risk category, the treatment also included fluoride and/or chlorhexidine varnish treatments four times a year. The reduction in total costs was mainly based on the role of the preventive dental assistants carrying out the programme. The time spent on prevention at an early age reduced the need for restorative treatment, and, thus, the clinical time of a dentist/assistant team [8,9].

Examinations and the examination intervals are of great interest to the public health care. It is a routine system in Finland that the public health care centre determines the time and frequency of dental examinations of all under 18-year-olds. The aim of the present study was to examine the results from the early targeted prevention programme in relation to clinical and economic findings from the perspective of public health care seven years after the cessation of the targeted programme which had been followed by generally applied preventive measures in Finnish public health care centres.

The article compares clinical and economic outcomes of a risk-based caries prevention programme with conventional prevention given in a neighbouring health centre, seven years after the 3-year programme was discontinued.

## Methods

### Subjects

The first phase of the study was carried out as a field study in the Public Health Care Centres of Vanha Korpilahti and Saarijärvi, in Central Finland. The risk-based prevention group in Vanha Korpilahti and the routine prevention group in Saarijärvi included all two-year-old children born in 1987 or 1988. These children were followed up until the 5-year examination [8]. At the first examination, written informed consent was obtained from the parents of the children in the risk-based prevention group. The Ethical Committees of the Medical Faculty of Turku University and the Public Health Care Centre in Vanha Korpilahti approved the original study protocol.

After the 5-year examination, the risk-based programme was discontinued. For the present study, the additional follow-up time was 7 years. The present material was collected from the dental records of the health care centres, and included all dental visits after the 5-year examination until the 12-year examination. The children had been examined regularly at individual intervals. The recommendations of the health care centre had included examination of all 12-year-olds. For the present study, the health care centres gave their permission to use the dental records.

### Drop-outs

In the first phase of the study, the parents of five subjects refused to allow their children to participate in the programme. During the 3-year follow-up, the percentage of drop-outs was 13% in the risk-based and 16% in the routine prevention group of the children examined at baseline, the main reason being moving away from the district. During the additional 7-year follow-up, the percentages of drop-outs were 18% and 11%, respectively, of the children examined at the age of 5 years.

### Clinical examinations

A mirror with 1.6 -fold magnification, a blunt periodontal probe and fibre-optic transillumination were routinely used. The examinations were carried out in a dental unit with good light and compressed air. X-rays were taken on an individual basis.

Tooth decay, fillings and sealants were registered at surface level in the dental records of the public health care centre. A tooth surface was considered sound when no sign of demineralisation was observed. Incipient caries lesions in enamel and early dentinal lesions when no cavity was present clinically, were coded "I". When a defect was found clinically on the surface and restorative treatment was considered necessary, the lesion was coded "D". At the age of 12 years, the total caries experience was expressed at tooth level using the DMF index, and at surface level using the I+DMFS index.

### Treatment given

After the subjects were 5 years of age no study-related recommendations were given to the dentists or the hygienists responsible for the dental care. The subjects received preventive and restorative treatment when the examining dentist considered it necessary. The prevention included dental health education, fluoride varnish treatments, sealants, and chlorhexidine treatments. The prevention was mainly delegated to dental hygienists or preventive dental assistants, but the dentists also gave preventive treatment in connection with other visits. Thirty minutes was the time reserved for each visit, regardless of the treatment or who carried it out.

At the age of 12 years, the number of dental visits after the 5-year examination was collected and classified: first, the visits to preventive dental assistants or hygienists, consisting only of examinations and/or prevention, and, second, the visits to a dentist/assistant team, separating those consisting mainly of preventive non-operative treatment and those consisting mainly of restorative treatment.

### Economic evaluation

The economic analysis was done from the perspective of a public health care centre. We used the actual running costs of the health care centre for the analyses. According to the annual report of Vanha Korpilahti Public Health Care Centre, in 1999, salary costs represented 73% of total running costs. In each personnel group, the hourly costs of clinical work were calculated by dividing the yearly salary of the group by their clinical working hours. The costs included the social security costs (31% of the salary), and they were calculated at 1999 rates. The total running costs (including materials, etc.) of the dentist/assistant team were estimated at 110 €/h. The equivalent costs of a preventive dental assistant and a hygienist were estimated at 43 €/h.

The costs per child for the 7-year follow-up period were calculated by multiplying the time spent by the costs per hour of the required personnel. This was carried out for preventive dental assistants or hygienists, and for a dentist/assistant team (separately for preventive and restorative treatment). The sum of these three costs represented the total running costs. The time and costs related to treatment of traumas or orthodontic treatment were not included in the present analyses. The salary costs of the routine prevention group were calculated using the same figures as above.

### Analysis of data

At the age of 12 years, the groups were compared in relation to clinical outcomes by using the DMF and I+DMFS indices, and the proportion of caries-free permanent dentitions (DMF = 0). In terms of use of clinical resources, the groups were compared using the number of sealants, the number of dental visits needed from 5 to 12 years of age, and the estimation of running costs during these years.

### Statistical analyses

Univariate analysis of variance was used to test the effect of group, risk category, and their interaction effect on DMF, I+DMFS, the number of sealants, and the cumulative number of visits at the age of 12 years, as well as on the estimated running costs from 5 to 12 years of age. If the interaction effect was significant (p < 0.05), the significance of group differences was tested using the t-test for independent samples, separately, in the three risk categories. The analyses were carried out using SPSS statistical software (SPSS, release 10.0.5 for Windows). The absolute risk reduction and NNT values were calculated according to Sackett et al. [10].

## Results

### Clinical outcomes

DMF at the age of 12 years was significantly related to the risk category determined ten years earlier (the presence of MS in plaque and/or incipient caries lesions at the age of 2 years as caries risk indicators). The level of caries was higher with higher estimated risk in both groups, although the level of caries was highly significantly lower in the earlier risk-based group than in the routine prevention group (Table 1).

**Table 1:** Number of DMF teeth, I+DMF surfaces, and sealants at the age of 12 years. Analysis of variance: DMF: Group effect, p < 0.001, Risk category effect, p < 0.001;I+DMFS: Group effect, not significant, Risk category effect, p < 0.001; Sealants: Group effect, p < 0.001, Risk category effect, p = 0.05

**Prevention group**		**DMF**		**I+DMFS**		**Sealants**	
Risk category at 2 yrs	*n*	Mean	S.D.	Mean	S.D.	Mean	S.D.
**Risk-based *N = 245***		**0.2**	**0.6**	**2.8**	**4.4**	**2.6**	**2.5**
Low-risk	*166*	0.2	0.6	2.4	3.8	2.4	2.5
Intermediate	*52*	0.2	0.7	3.1	4.7	2.8	2.2
High-risk	*27*	0.4	0.8	4.7	6.4	3.4	2.9

**Routine *N = 202***		**0.4**	**0.9**	**2.9**	**4.0**	**1.6**	**1.8**
Low-risk	*134*	0.3	0.7	2.3	3.8	1.5	1.6
Intermediate	*50*	0.6	0.9	4.1	3.8	1.5	1.8
High-risk	*18*	0.9	1.4	4.6	4.7	2.2	2.9

When incipient caries lesions were included in the analyses the association between caries at the age of 12 years (I+DMFS) and risk category at the age of 2 was highly significant (Table 1). The group effect was not significant.

At the age of 12 years, the proportion of permanent dentitions with caries was 0.13 in the risk-based and 0.26 in the routine prevention group. The absolute risk reduction for caries was thus 0.13 (95% confidence interval 0.06 – 0.21), and the associated NNT value was 8 (95% confidence interval 5 – 17).

At the age of 12 years, the frequency of sealants was higher with higher estimated risk in both groups. The number of sealants was significantly higher in the earlier risk-based group than the routine prevention group (Table 1).

### Dental visits

The total number of visits to a dental surgery from 5 to 12 years of age was associated with the risk category determined at the age of 2 years; the number of visits being higher with higher risk (Table 2). In the earlier risk-based group, the dentists gave less, and the preventive dental assistants or hygienists clearly more preventive treatment than in the routine prevention group. Altogether, the number of preventive visits was lower in the earlier risk-based than in the routine prevention group.

**Table 2:** Number of visits for dental treatment from 5 to 12 years of age. Analysis of variance: Preventive/Dental hygienist: Group effect, p = 0.002, Risk category effect, p < 0.001; Preventive/ Dentist – dental assistant team: Group effect, p < 0.001; Restorative/ Dentist – dental assistant team: interaction term Group*Risk category, p = 0.001, Group difference: low-risk p < 0.09, intermediate p = 0.003, and high-risk category p < 0.03

**Prevention group**		**Dental hygienist**		**Dentist-dental assistant team**			
				
		**Preventive**		**Preventive**		**Restorative**	
Risk category at 2 yrs	*n*	Mean	S.D.	Mean	S.D.	Mean	S.D.
**Risk-based *N = 245***		**8.2**	**4.0**	**3.9**	**2.3**	**2.0**	**2.9**
Low-risk	*166*	7.5	3.6	3.9	2.4	1.8	2.6
Intermediate	*52*	8.8	3.9	4.0	2.1	2.4	3.4
High-risk	*27*	11.6	4.2	3.9	2.6	2.7	3.6

**Routine *N = 202***		**7.2**	**2.6**	**5.7**	**1.5**	**3.4**	**4.4**
Low-risk	*134*	6.5	2.3	5.5	1.5	2.4	3.4
Intermediate	*50*	8.6	2.3	5.8	1.6	4.8	4.5
High-risk	*18*	8.9	3.3	6.3	1.1	7.1	7.4

The number of visits because of restorative treatment during the seven-year follow-up was associated with the risk category determined at the age of 2 years (Table 2). There were significant differences in the number of restorative visits between the groups in the intermediate and high-risk categories (Table 2).

### Running costs

**Figure 1:**
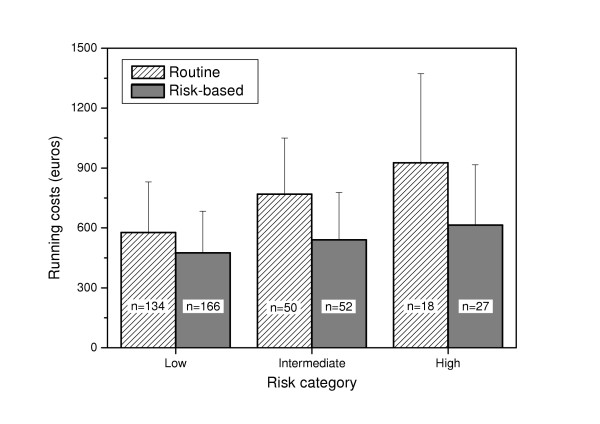
Total running costs (mean and standard deviation) from 5 to 12 years of age. Risk categories were formed on the basis of information on MS and incipient caries at the age of 2. The figure within the column refers to the number of subjects within the category. Analysis of variance: Interaction term Group*Risk category, p = 0.009 Group difference: Low-risk category, p < 0.001; Intermediate category, p < 0.001; High-risk category, p = 0.02

On average, the estimated running costs were lower in the earlier risk-based group (mean 505 €; S.D. 230) than in the routine prevention group (mean 656 €; S.D. 304). The difference was significant in all risk categories (Figure 1). The components of the costs are shown in Table 3.

**Table 3:** Mean running costs (in €) related to dental treatment from 5 to 12 years of age.

**Prevention group**		**Dental hygienist**		**Dentist- dental assistant team**			
				
		**Preventive**		**Preventive**		**Restorative**	
Risk category at 2 yrs	*n*	Mean	S.D.	Mean	S.D.	Mean	S.D.
**Risk-based *N = 245***		**177**	**86**	**216**	**129**	**111**	**160**
Low-risk	*166*	161	79	215	132	99	142
Intermediate	*52*	190	84	220	114	130	188
High-risk	*27*	251	90	214	143	149	200

**Routine *N = 202***		**156**	**56**	**312**	**84**	**188**	**244**
Low-risk	*134*	140	49	305	85	133	188
Intermediate	*50*	186	50	319	88	263	249
High-risk	*18*	192	72	346	59	388	406

## Discussion

The findings indicate that early risk-based prevention can be correctly targeted, clinically effective, and of economic benefit, also from the long-term perspective. This is in line with earlier studies among young children [11,12]. It seems to be possible to predict the first caries attack with acceptable accuracy, and also to succeed in the prevention itself with reduced costs. This is in contrast with several studies, which have without success tried to find an accurate method to identify risk subjects among schoolchildren or adolescents [13,14]. Even with relatively successful identification, the applied prevention does not suggest good results in these age groups [15].

Clinical outcomes indicated a clear association between caries level at the age of 12 years and the risk category determined at the age of 2 years. This association was found both in the risk-based study group and in the control group with conventional prevention. At the age of 12 years, in the risk-based group, a lower level of caries experience in permanent teeth was observed than in the routine prevention group, which indicated that the risk-based prevention had been correctly targeted throughout the years.

The clinical effectiveness of early dental health education has been demonstrated in immigrant-background families in low socio-economic/high caries suburbs of Leeds in the United Kingdom [16]. The present study showed that even in subjects in communities with very low average caries occurrence figures, the early preventive measures led to increased prevention.

In terms of population and economic structure, Saarijärvi and the three communities within the Vanha-Korpilahti public heath care centre catchment area were comparable. The soil in the area contains a low level of fluoride. Almost the whole population in both communities was included in the dental recall system within the public health care centres. After the subjects in the present study reached the age of 5 years, there was no strict programme concerning the dental preventive non-operative or restorative treatment in either health care centre. Thus, the first difference between the risk-based and the routine prevention groups was the screening and prevention programme introduced from 2 to 5 years. However, it is possible that the differences in the prevention programmes during 2 to 5 years of age are associated with the later differences in the measures used, as can be seen in the high number of sealants used in the risk-based group (Table 1). Therefore, a part of the result may be based on these differences between 5 and 12 years of age.

Work division in dentistry is currently being hotly debated in Finland in an effort to find optimal cost-benefit ratios. The total clinical time used during the years 2–5 was the same for both the study and the control health care centres, but it was distributed unevenly. In Vanha-Korpilahti, more time was spent on prevention carried out by the hygienists, but less time on treatment calling for the dentist – dental assistant teams. Therefore, the economic results were mainly based on the work division. The second clear difference between the groups was in the structure of the personnel: in Vanha-Korpilahti, there were five hygienists and one preventive dental assistant for five dentist/assistant pairs, while in Saarijärvi, there was only one hygienist for every six dentist /assistant pairs.

The salary costs represent the major proportion of the total costs. We did not discount the costs. Because the costs were lower in Vanha-Korpilahti than in Saarijärvi when the subjects were 2–5 years, the discounting had slightly increased the difference between the health care centres in favour of the risk-based targeted prevention.

The costs related to parents and their children were not included. The time the parents and children spent on the programme is closely related to the number of dental visits, but not as much to the length of the visits. The present results should not be interpreted in such a way that all work division in prevention is effective if applied mechanically. If a child is already sitting in the dental chair for diagnosis, treatment etc., it is usually more practical for the dentist-dental assistant team to carry out the preventive clinical measures during the same visit. This saves a lot of time in the change-over of patients, and also in instrument maintenance.

## Conclusion

Early prevention of dental caries has long-term benefits. This seems to hold true as regards targeting, clinical and economic effectiveness also from a follow-up perspective. Success in risk-based prevention allows successful work division, and consequently, economic effectiveness.

## List of abbreviations

DMF the number of decayed, missed or filled teeth

I the number of incipient caries lesions

MS mutans streptococci

NNT number needed to treat

## Competing interests

The author(s) declare that they have no competing interests.

## Authors' contributions

KP participated in the design of the study, performed the statistical analyses, and drafted the manuscript. JJ participated in the design of the study, and collected the data at the health care centre. PA participated in the design of the study and in writing the manuscript. All authors read and approved the final manuscript.

## Pre-publication history

The pre-publication history for this paper can be accessed here:


